# Impact of comprehensive geriatric assessment on survival, function, and nutritional status in elderly patients with head and neck cancer: protocol for a multicentre randomised controlled trial (EGeSOR)

**DOI:** 10.1186/1471-2407-14-427

**Published:** 2014-06-13

**Authors:** Lydia Brugel, Marie Laurent, Philippe Caillet, Anne Radenne, Isabelle Durand-Zaleski, Michel Martin, Melany Baron, Héloïse de Kermadec, Sylvie Bastuji-Garin, Florence Canouï-Poitrine, Elena Paillaud

**Affiliations:** 1 Centre Hospitalier Intercommunal de Créteil, Service d’ORL et Chirurgie Cervico-faciale, Créteil F-94010, France; 2 AP-HP, hôpital Henri-Mondor, Département de Médecine Interne et Gériatrie, Unité d’Onco-Gériatrie, Créteil F-94010, France; 3 Université Paris Est Créteil (UPEC), LIC EA 4393, Créteil F- 94010, France; 4 AP-HP, hôpital Henri-Mondor, Unité de Recherche Clinique (URC-Mondor), Créteil F-94010, France; 5 URCEco Ile-de-France, Hôpital de l’Hotel Dieu, Paris F-75004, France; 6 Centre Hospitalier Intercommunal de Créteil, Service d’Oncologie médicale, Créteil F-94010, France; 7 Centre Hospitalier Intercommunal de Creteil, Service de Gériatrie, Créteil F-94010, France; 8 AP-HP, hôpital Henri-Mondor, Service de Santé Publique, Créteil F-94010, France

**Keywords:** Comprehensive geriatric assessment, Head and neck cancer, Elderly patients

## Abstract

**Background:**

Survival is poorer in elderly patients with head and neck squamous cell carcinomas [HNSCCs] than in younger patients. Possible explanations include a contribution of co-morbidities to mortality, frequent refusal of standard therapy, and the use of suboptimal treatments due to concern about toxicities. The Comprehensive Geriatric Assessment [CGA] is a multidimensional assessment of general health that can help to customise treatment and follow-up plans. The CGA has been proven effective in several health settings but has not been evaluated in randomised studies of patients with cancer. Our aim here was to assess the impact of the CGA on overall survival, function, and nutritional status of elderly patients with HNSCC.

**Methods/design:**

EGeSOR is an open-label, multicentre, randomised, controlled, parallel-group trial in patients aged 70 years or older and receiving standard care for HNSCC. The intervention includes four components: the CGA conducted by a geriatrician before cancer treatment, participation of the same geriatrician in cancer treatment selection, a standardised geriatric therapeutic intervention designed by the same geriatrician; and geriatric follow-up for 24 months. The primary endpoint, assessed after 6 months, is a composite criterion including death, functional impairment [Activities of Daily Living score decrease ≥2], and weight loss ≥10%. Secondary endpoints include progression-free survival, unscheduled admissions, quality of life, treatment toxicities, costs, and completion of the planned cancer treatment. A centralised online system is used to perform 1:1 randomisation with a minimisation algorithm for centre, age, T and N stages, and tumour site [oral, oropharyngeal, hypopharyngeal, or laryngeal]. The estimated sample size is 704 patients, who are being recruited by 14 centres in 9 French cities.

**Discussion:**

EGeSOR is the first randomised trial of the CGA in elderly cancer patients. We expect the CGA to have direct clinical benefits on the management of elderly patients with HNSCC. If this expectation is fulfilled, the trial may lead to modifications of the management model for elderly patients with cancer.

**Trial registration:**

Trial registration: NCT02025062

## Background

Head and neck cancers are the sixth most common cancer in the world according to 2006 European Cancer Observatory data [1]. In parallel with the rise in life expectancy, the number of elderly patients with head and neck squamous cell carcinomas [HNSCCs] is increasing, especially in women [2,3]. Overall survival [OS] in patients with HNSCCs has been estimated at about 50% after 5 years, with large variations across tumour sites [4-6]. Two studies suggest lower 5-year OS rates in patients aged 75 or over than in younger patients [7,8]. Possible explanations to this difference may include a contribution of co-morbidities to mortality [7,9], greater patient reluctance to undergo full treatment regimens, and physician choice of suboptimal treatments due to concern about toxicities [10]. Co-morbidities become increasingly prevalent with advancing age and are associated with treatment-related side effects and poorer outcomes [9,11-13]. Although studies support the use of similar cancer treatments in older and younger patients [14,15], a thorough pre-treatment evaluation is deemed crucial, most notably in elderly patients [16-18].

The Comprehensive Geriatric Assessment [CGA] was developed by geriatricians as a ‘multidimensional interdisciplinary diagnostic process focussed on determining a frail older person’s medical, psychological, and functional ability in order to develop a coordinated and integrated plan for treatment and long-term follow-up’ [19]. The CGA relies on validated geriatric scales or tests to draw a detailed picture of the patient’s health status, which can then serve to develop an individualised geriatric intervention plan. The CGA is therefore both a diagnostic and a therapeutic tool. It is designed to ensure that all problems are identified, quantified, and managed appropriately. A meta-analysis showed that the CGA, combined with multidisciplinary interventions, improved survival and function and decreased the need for admission and institutionalisation in elderly patients with non-malignant diseases [20]. Over the past decade, the CGA has been suggested for elderly patients with cancer and recommended by the International Geriatric Oncology Society [SIOG] [21] as a means of optimising cancer treatment selection, improving the chances of treatment completion, increasing survival, and diminishing adverse outcomes [22-24]. However, randomised trials of the CGA are available only for non-malignant conditions: there is no high-level evidence on the potential benefits of the CGA in elderly patients with cancer.

We hypothesised that performing the CGA in elderly patients with HNSCC would improve treatment decision-making by better evaluating the patient’s functional reserve, a crucial factor in the ability to tolerate cancer treatments; and would improve OS, function, and nutritional status by ensuring optimal customisation of the treatments and follow-up during surgery and/or radiotherapy and/or chemotherapy. Our objective was to evaluate the impact of the CGA on OS, function, and nutritional status of elderly patients with HNSCC.

## Methods/design

### Study design and setting

**Figure 1:**
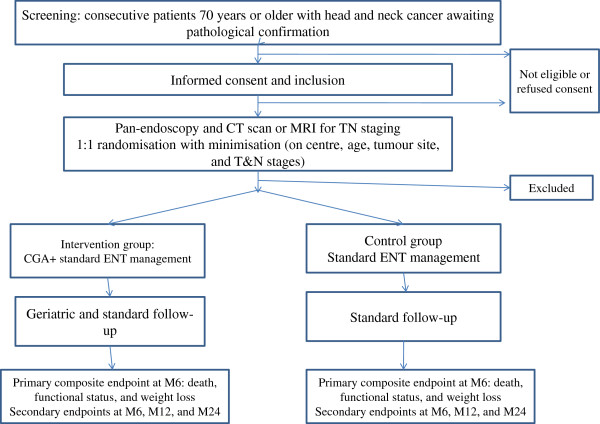
EGeSOR flow chart.

We are conducting an open-label, multicentre, parallel-group, randomised, controlled trial in patients aged 70 years or older and receiving standard care for HNSCC. One group receives a CGA-based multi-component intervention and the other does not [Figure 1]. The primary endpoint is a composite criterion to be assessed after 6 months. Other endpoints such as OS and disease-free survival are assessed after 24 months. The patients are being recruited at 14 ENT/maxillo-facial surgery departments in 13 hospitals located in 9 cities in France [Paris, Créteil, Montfermeil, Villeneuve-Saint-Georges, Lille, Strasbourg, Nancy, Nantes, and Suresnes]. The protocol was approved by the appropriate ethics committee [CPP Ile-de-France I, Paris, France, approval April, the 20 April 2013;13213]. The trial is registered on ClinicalTrials.gov [NCT02025062].

### Study population

Inclusion criteria are age ≥70 years; macroscopic diagnosis of head and neck cancer [oral, oropharyngeal, hypopharyngeal, or laryngeal] awaiting histological confirmation; coverage by the French statutory health insurance system; and written informed consent obtained from the patient. Non-inclusion criteria are as follows: correctional facility inmate; legal guardianship; psychological, familial, social, or geographic conditions that might interfere with the conduct of the study; personal history of head and neck cancer; and rare tumour site [sinonasal or salivary gland]. To prevent selection bias limiting the general applicability of our findings, we are including consecutive patients who meet the eligibility criteria.

### Study intervention

The study intervention has four components.

(a) CGA before cancer treatment initiation, performed by a geriatrician [designated the ‘intervention geriatrician’ hereafter];

(b) participation of the intervention geriatrician in developing the cancer-treatment plan;

(c) standardised multidimensional geriatric therapeutic programme designed by the intervention geriatrician; and

(d) geriatric follow-up during cancer treatment and for 24 months after randomisation.

#### (a) CGA

The CGA is performed by the intervention geriatrician, after randomisation and before the multidisciplinary meeting during which the cancer-treatment plan is established. The CGA consists in a detailed medical, psychological, and social assessment including a careful medical history and physical examination. In accordance with SIOG recommendations for elderly cancer patients, seven domains are assessed routinely: functional status, mobility and fall risk, nutritional status [25], cognitive status, mood, co-morbidities and medications, and social environment. These domains are assessed using validated tests, questionnaires, scores, and/or scales [Table 1]. Pain is also evaluated using a verbal numeric scale. The CGA is performed by a senior geriatrician, in some cases with help from a trained healthcare worker [usually a nurse working in the oncology and/or geriatric department].

**Table 1:** Primary and secondary endpoints in the EGeSOR trial

**Endpoints**	**Time assessed**	**Description**
Primary endpoint	6 months after randomisation	Composite criterion including
		- death,
		- at least 2-point decrease in the Activities of Daily Living (ADL) score versus baseline
		- at least 10% decrease in body weight versus baseline
Secondary endpoints	6, 12, and 24 months after randomisation	- each component of the primary endpoint
		- progression-free survival
		- in-hospital death
		- unplanned admissions
		- post-surgery hospital stay length
		- discharge to home or nursing home
		- final cancer treatment plan (surgery, chemotherapy, targeted therapies, radiotherapy, and/or supportive care, alone or combined)
		- quality of life assessed by EORTC QLQ-C30 and specific module for head and neck cancer H&N35
		- treatment toxicities and/or complications: chemotherapy toxicities according to Classification Common Terminology Criteria for Adverse Events (CTCAE version 4.02)
		- cancer treatment feasibility
		- costs

#### (b) Geriatrician participation in development of the cancer-treatment plan

The intervention geriatrician lists all identified problems by order of priority and provides specific advice on the treatment goal [curative or palliative] and modalities [surgery, radiotherapy, chemotherapy, targeted therapy, and supportive care, used sequentially or simultaneously] in a written report given to the ENT physicians in charge of cancer treatment. The intervention geriatrician then participates in the multidisciplinary meeting held to determine the cancer-treatment plan [23].

#### (c) A standardised multidimensional geriatric therapeutic programme

A geriatric therapeutic programme is implemented at baseline and during follow-up. The intervention geriatrician designs this programme based on the CGA findings, in cooperation with the ENT physicians, other ENT healthcare staff, and outpatient healthcare professionals. The programme has four components: optimising the management of problems detected in the seven health domains evaluated during the CGA, a medication review, patient education on co-morbidity self-management, and information on cancer treatments [26]. These components are standardised and compliant with the most recent recommendations issued by the French National Authority for Health [HAS], French Society for Gerontology and Geriatrics, and French Society for Cardiology, as appropriate [Table 2]. Regarding co-morbidities, the geriatric intervention focuses on the five most common conditions present in elderly patients with head and neck cancer: chronic atrial fibrillation, chronic heart failure, diabetes, coronary artery disease, and hypertension. Corrective measures are taken as required. A geriatrician specialised in geriatric oncology [PC] is available by phone or e-mail for discussion with the intervention geriatrician. Careful attention is directed to complaints of pain. Analgesic medications are adjusted as needed and patients referred to a pain clinic if appropriate.

**Table 2:** Standardised multidimensional geriatric therapeutic programme in the intervention arm of the EGeSOR trial; ADL, Activities of Daily Living score; IADL, Instrumental Activities of Daily Living score; MNA, Mini-Nutritional Assessment; BMI, body mass index; MMSE, Mini-Mental State Examination; GDS, Geriatric Depression Scale; CIRS-G, Cumulative Illness Rating Scale-Geriatrics

**Domains**	**Assessment tools – thresholds for interventions**	**Standardised responses**
Functional status	ADL ≤5	Social services notification
	AND/OR	Home care
	IADL ≤ 7	Access to geriatric network
Mobility/fall risk	Falls during the last 6 months	Walking and/or standing-balance rehabilitation (20 sessions)
	AND/OR	AND
	One-leg standing test <5 seconds	Fall management^(1)^
	AND/OR	
	Timed get-up-and-go test >20 seconds	
Nutritional status	MNA ≤ 17	Nutritional care according to severity of malnutrition and swallowing disorders^(2)^
	AND/OR	- Dietician visits
	Weight loss ≥5% in the last 3 months	- High-energy and high-protein diet
	AND/OR	- Nutritional supplements
	Weight loss ≥10% in the last 6 months	- Enteral nutrition
	AND/OR	- Monitoring of local/regional treatment prescribed by the ENT physician, including oral care^(3)^
	BMI < 21 Kg/m^2^	- Access to geriatric network
		- Education on disease self-management
Cognitive status	MMSE ≤ 23	- Evaluation for causes of delirium and correction of predisposing factors^(4)^
		- Neuropsychological assessment with evaluation of memory
		- Access to geriatric network
Depression	GDS-15 ≥ 6	- Antidepressant treatment^(5)^
		- Follow-up by a psychologist
		- Psychiatrist visit, depending on severity
		- Access to geriatric network
Co-morbidities	CIRS-G:	- Medication review and medication regimen optimisation^(6)^
	at least one co-morbidity (other than the HNSCC) grade ≥3	- Access to geriatric network
	AND/OR	- Education on disease self-management:
	Number of drugs ≥5/day	- Diabetes in the elderly: facts and management^(7)^ - Atrial fibrillation: facts and management^(8)^
	Focus on five diseases: chronic atrial fibrillation, chronic systolic heart disease, diabetes, coronary artery disease, hypertension	- Management of coronary heart disease in older adults^(9)^
		- Diagnosis and management of chronic systolic heart disease

^(1)^Assessment and prevention of falls in older people – Practice guidelines. Online in April 2009.

^(2)^Malnutrition in the elderly - Nutritional support strategy. Practice guidelines. Online in June 2007.

Physiotherapy - Preserving motor function in frail elderly people living at home Practice guidelines. Online in April 2005.

^(3)^Upper aerodigestive cancers - Practice guidelines ALD n°30. Online in November 2009.

^(4)^Alzheimer's disease and related conditions - Diagnosis and treatment: Practice guidelines. Online in December 2011.

^(5)^A workshop on psychotropic drug prescriptions in the elderly. Online in October 2007.

^(6)^Improving the primary care prescription of hypnotic and anxiolytic drugs in the French elderly. Online in March 2009.

^(6)^Improving drug prescription in older persons. Online in November 2012.

^(7)^Guidelines for the management and care of diabetes in the elderly. Online in January 2011.

^(8)^Guidelines for the management of patients with atrial fibrillation. Online in July 2007.

All guidelines available at http://www.has-sante.fr/portail/jcms/fc_1249588/fr/accueil-2012/ Last accessed 27 December 2013.

Expert consensus of the French Society for Geriatrics and Gerontology and French Society for Cardiology on the management of atrial fibrillation in elderly people (2013), at http://www.sfcardio.fr/.

^(9)^ Consensus of the French Society for Gerontology and Geriatrics and French Society for Cardiology for the management of coronary heart disease, at http://www.sfcardio.fr.

^(10)^ Therapeutic education in patients with chronic heart failure : proposal for multiprofessional structured programme by a French task force under the auspices of the French Society of Cardiology at http://www.sfcardio.fr.

#### (d) Geriatric follow-up

**Figure 2:**
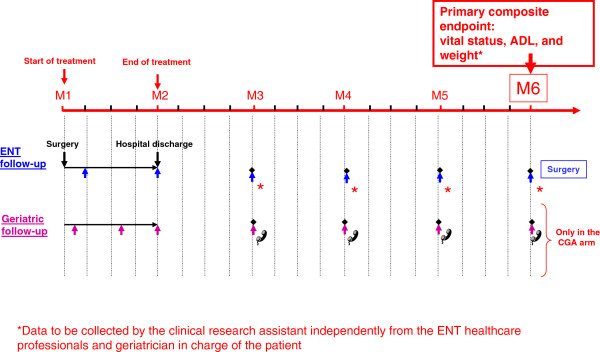
Follow-up over the first 6 months (M) for patients treated with surgery alone in the EGeSOR study.

The standardised geriatric follow-up provided by the intervention geriatrician, consists in a brief assessment of nutrition, mood, pain, functional status, the five above-listed co-morbidities, self-perceived health status, medication use, and implementation of the multidimensional geriatric therapeutic programme. The results of this standardised follow-up are used to make recommendations to the ENT physician, oncologist, radiotherapist and general practitioner.Follow-up includes closely spaced physical examinations for 1 to 6 months depending on the treatment modalities; Figure 2 is an example for a surgically managed patient. After completion of the initial cancer treatment, the intervention geriatrician or nurse phones the patient every 3 months during the first 2 years after randomisation. If problems are identified during any of these phone calls, an appointment for a visit with the intervention geriatrician is scheduled.

During follow-up, the patient has access to the resources provided by the local geriatric network: geriatric rehabilitation unit, geriatric day hospital, geriatric and/or oncology community centre, geriatric nursing home intervention, and home nursing care.

### Intervention geriatrician

The intervention geriatricians involved in the study are senior geriatricians with a mean of 3 years of post-degree experience in geriatrics. Before inclusion of the first patient in their centre, the EGeSOR coordination team composed of four geriatricians [EP, PC, ML, MB] provided the intervention geriatricians with a half day of training in the EGeSOR intervention and in the specific features of HNSCC.

### Endpoints

Table 2 shows the primary and secondary endpoints. The primary endpoint is a composite criterion evaluated 6 months after randomisation and including death, an at least 2-point decrease in the Activities of Daily Living [ADL] score [27] versus baseline, and at least 10% weight loss versus baseline. A clinical research assistant blinded to the randomisation arm uses a standardised measurement guide to adjudicate the ADL score and weight. Weight is measured to the nearest 0.1 Kg using an electronic scale [Seca Model 876, Birmingham, United Kingdom]. Our decision to include functional and nutritional measures into the primary endpoint rests both on sound evidence that these measures influence treatment feasibility, patient survival, and quality of life; and on the potential for appropriate management to reverse functional impairments and weight loss.

### Random assignment

After including each patient and obtaining the panendoscopy and computed tomography or magnetic resonance imaging findings to allow TN staging, the ENT physician or clinical research assistant records the centre, patient age, tumour site, and TN stage into an online central randomisation system [RandoWeb, Paris, France] [28]. The software automatically checks the data for completeness and consistency then allocates the patient to the intervention group or control group. All patients receive standard HNSCC therapy. The randomisation system applies a minimisation programme to balance the two groups regarding centre, age [<or ≥80 years], T stage [<or ≥ T2], N stage [<or ≥ N2], and tumour site [oral, oropharyngeal, hypopharyngeal, or laryngeal]. Minimisation randomly allocates the first patient to a group then allocates each subsequent patient to the group that produces the smallest difference between treatment groups regarding the five above-listed factors [29]. Because simple minimisation within centres can, in theory, lead to alternation of treatment allocation, the algorithm also incorporates 30% of random allocation. This helps to ensure concealment.

### Sample size estimation

We hypothesised that the intervention would result in an at least 10% absolute decrease in the primary endpoint, and we assumed that 30% of controls would achieve the primary endpoint. With a 5% two-sided alpha risk and 80% power, 640 patients would be needed [320 in each group]. We assumed that 10% of patients would be lost to follow-up before study completion or would not have data on the primary endpoint. Therefore, we plan to include 704 patients in all.

### Statistical analysis

Data will be analysed according to CONSORT guidelines [30]. The descriptive analysis will compare the two randomised groups in terms of general characteristics, demographics, co-morbidities, risk factors, and baseline HNSCC characteristics. Only descriptive statistics will be used at this stage. Quantitative variables will be described as mean [±1 standard deviation (SD)] or median [25-75th percentiles] according to their distribution and qualitative variables as numbers [%].

A flow diagram will be created according to CONSORT guidelines. The primary endpoint will be analysed using the intent-to-treat approach, with all patients kept in the group to which they were assigned by the randomisation system. We will compare the proportions of patients achieving the primary endpoint in the two groups using Pearson’s chi-square test, and we will assess effect size by computing the relative risk with its 95% confidence interval [95% CI] and the absolute difference between groups. This analysis will be adjusted for any baseline patient characteristics that are imbalanced between the two groups. For between-group comparisons of secondary endpoints, we will use the same method as for the primary endpoint.

Variables measured serially during follow-up [functional status, depressive mood, weight, pain, and quality of life] will be analysed using mixed models to take into account the repetition of the measures. Quality-of-life data will be analysed using QLQ-C30 and H&N35 [8,31,32]. Subgroup analyses will be conducted according to age group [<or ≥80 years], tumour site, and tumour stage.

### Costs

We will estimate the cost of our four-component intervention, compare the costs of the intervention and control management strategies, and compute an incremental cost-effectiveness ratio. Costs will be estimated from the perspective of the French healthcare system, from randomisation to death or end of follow-up. Only direct costs will be counted. In each patient, all resources used will be recorded prospectively, using the same methodology in both groups. Resources used include the CGA [physician visits, medical interventions, and investigations], treatments [surgery, cytotoxic or targeted chemotherapy, radiotherapy, and supportive treatments (including recombinant human erythropoietin, anti-emetics, and colony-stimulating factors)], nutritional care [nutritional supplements and enteral nutrition], acute admissions for any reason, residence in a nursing home or rehabilitation unit, community care [physician visits and investigations during follow- up, number of physiotherapist sessions, number of home healthcare professional visits, and care provided at skilled nursing facilities]. The cost per resource unit will be taken from the cost lists established by the French statutory healthcare system. Differences in costs and differences in effectiveness on the primary endpoint will be used to estimate the incremental cost-effectiveness ratio. Costs will be reported as median [25th-75^th^ percentiles] and mean [SD] and compared using the Wilcoxon-Mann–Whitney test and parametric tests. Confidence intervals will be estimated by bootstrapping with 1000 re-samplings of the original dataset.

The significance level will be 5%. All tests will be two-tailed. Data will be analysed using Stata Software [College Station, TX, USA].

## Discussion

The EGeSOR trial aims to demonstrate that a multidimensional geriatric intervention based on an initial CGA, when added to standard care for HNSCC in elderly patients, significantly improves a 6-month composite endpoint of survival, functional status, and weight. The full picture of co-morbid conditions produced by the CGA may improve cancer treatment selection. Many elderly individuals have potentially fatal co-morbidities, which both compete with HNSCC as a cause of death and influence the risk/benefit ratio of cancer treatments. Moreover, the data on functional reserve provided by the CGA may help to predict the patient’s ability to tolerate specific cancer treatments. The personalised geriatric follow-up during cancer treatment, including adjustment of the treatments and management of the co-morbidities and iatrogenic complications, may increase the likelihood of cancer-treatment feasibility, improve quality of life, and decrease unplanned admissions and hospital stay length. Finally, the trial preparation phase strengthened the collaboration between ENT physicians and geriatricians, and we expect this beneficial effect to continue throughout the trial.

The study intervention consists of four components: the CGA performed by the intervention geriatrician at baseline, participation of the intervention geriatrician in developing the cancer treatment plan, a standardised multidimensional geriatric therapeutic programme, and geriatric follow-up for 24 months. The first two components have been proven to benefit treatment decisions [23,33-35]. A Cochrane Collaboration meta-analysis showed that the last two components improved survival and maintenance at home in elderly patients with non-malignant diseases [20]. The SIOG has recommended performing the CGA in vulnerable elderly patients with cancer [21]. However, no previous studies evaluated the impact of the CGA in elderly patients with cancer.

The EGeSOR trial has several limitations. First, the CGA is time consuming and requires extensive involvement of geriatric teams, which may not be available in some centres. We visited each of the study centres to verify that the number of geriatricians was sufficient, and we considered reinforcing the geriatric team if necessary. Second, the primary endpoint is a composite criterion. However, demonstrating superiority of the study intervention on a single endpoint would have required such a large sample size as to compromise feasibility of the trial. Third, contamination bias may occur between the intervention and control groups, as each ENT physician manages patients in both groups. Conceivably, ENT physicians may apply knowledge acquired by managing intervention patients to their control patients, for instance by asking geriatricians for advice regarding vulnerable controls. Such contamination bias would diminish the size of the effect of the intervention. However, any contamination bias is probably limited, since modifications potentially introduced by ENT physicians in controls would have a very small effect compared to the full four-component intervention. Nevertheless, we are recording the number of times that advice from geriatricians is provided for control patients. Our study had several strengths. To our knowledge, it is the first randomised controlled trial evaluating the efficacy of the CGA in elderly cancer patients. We decreased the risk of selection bias by using randomisation with minimisation, the risk of evaluation bias by selecting objective clinical endpoints and using independent adjudication, and the risk of confusion bias by using randomisation with minimisation and a pre-specified multivariate analysis to correct for any baseline between-group imbalances. Finally, the standardised multidimensional geriatric therapeutic programme was designed in accordance with the most recent clinical recommendations and relied on validated international measurement tools.

## Conclusion

The CGA has been proven in several randomised trials and meta-analyses in the general geriatric population to improve survival, institutionalisation rates, and functional status. The EGeSOR trial is the first randomised trial of the CGA in elderly cancer patients. We expect to demonstrate a direct clinical benefit of the CGA on outcomes of elderly patients with HNSCC. If such a benefit is found, the results of the EGeSOR trial may change the healthcare management model for elderly patients with cancer.

## Abbreviations

ADL: Activities of daily living; CGA: Comprehensive geriatric assessment; ENT: Ear nose throat; HNSCC: Head and neck squamous cell carcinomas; ICER: Incremental cost-effectiveness ratio; OS: Overall survival; SIOG: International geriatric oncology society.

## Competing interests

The authors declare that they have no competing interests.

## Authors’ contributions

EP, LB, FCP and AR designed and managed the study. EP and FCP wrote the main part of the manuscript. The manuscript was critically revised by LB, PC, AR, and SBG. All authors read and approved the final manuscript. EP, LB, and FCP obtained the funding.

## Pre-publication history

The pre-publication history for this paper can be accessed here:

http://www.biomedcentral.com/1471-2407/14/427/prepub
